# *Schistosoma mansoni* venom allergen-like protein 6 (SmVAL6) maintains tegumental barrier function

**DOI:** 10.1016/j.ijpara.2020.09.004

**Published:** 2021-03

**Authors:** Samirah Perally, Kathrin K. Geyer, Priscila S.G. Farani, Iain W. Chalmers, Narcis Fernandez-Fuentes, Daniel R. Maskell, Benjamin J. Hulme, Josephine Forde-Thomas, Dylan Phillips, Leonardo P. Farias, James J. Collins, Karl F. Hoffmann

**Affiliations:** a Institute of Biological, Environmental and Rural Sciences (IBERS), Aberystwyth University, Aberystwyth SY23 3DA, United Kingdom; b Instituto Gonçalo Moniz, Fundação Oswaldo Cruz (FIOCRUZ), Rua Waldemar Falcão, Salvador, Bahia, Brazil; c Department of Pharmacology, University of Texas Southwestern Medical Center, Dallas, TX 75390-9041, USA

**Keywords:** Venom allergen-like, *Schistosoma mansoni*, Tegument

## Abstract

The *Schistosoma mansoni* venom allergen-like protein (SmVAL) superfamily is a collection of at least 29 molecules that have been classified into two distinctive groups (Group 1 and Group 2 SmVALs). The fundamental basis for SmVAL segregation relates to signal peptide and conserved cysteine retention (present in all Group 1 SmVALs, but absent in all Group 2 SmVALs). These structural differences have led to the hypothesis that most Group 1 SmVALs, found as components of schistosome excretory/secretory (E/S) products, predominantly interact with their environment (intermediate or definitive hosts) whereas the Group 2 SmVALs are retained within the schistosome to fulfil parasite-related functions. While experimental evidence to support Group 1 SmVAL/host interactions is growing, similar support for identification of parasite-related Group 2 SmVAL functions is currently lacking. By applying a combination of approaches to the study of SmVAL6, we provide the first known evidence for an essential function of a Group 2 SmVAL in schistosome biology. After whole mount in situ hybridisation (WISH) localised *Smval6* to the anterior region of the oesophageal gland (AOG) and cells scattered through the mesenchyme in adult schistosomes, short interfering RNA (siRNA)-mediated silencing of *Smval6* was employed to assess loss of function phenotypes. Here, si*Smval6-*mediated knockdown of transcript and protein levels led to an increase in tegumental permeability as assessed by the quantification of TAMRA-labelled dextran throughout sub-tegumental cells/tissues. Yeast two hybrid screening using SmVAL6 as a bait revealed Sm14 (a fatty acid binding protein) and a dynein light chain (DLC) as directly interacting partners. Interrogation of single-cell RNA-seq (scRNA-seq) data supported these protein interactions by demonstrating the spatial co-expression of *Smval6*/*dlc*/*Sm14* in a small proportion of adult cell types (e.g. neurons, tegumental cells and neoblasts). In silico modelling of SmVAL6 with Sm14 and DLC provided evidence that opposing faces of SmVAL6 were likely responsible for these protein/protein interactions. Our results suggest that SmVAL6 participates in oesophageal biology, formation of higher order protein complexes and maintenance of tegumental barrier function. Further studies of other Group 2 SmVALs may reveal additional functions of this enigmatic superfamily.

## Highlights

• *Smval6* is expressed in oral/ventral suckers, oesophageal gland and mesenchymal cells of *Schistosoma mansoni*.
• *Smval6* knockdown increases surface membrane permeability.
• SmVAL6 interacts with Sm14 and DLC proteins.

## Graphical abstract

**Figure:**
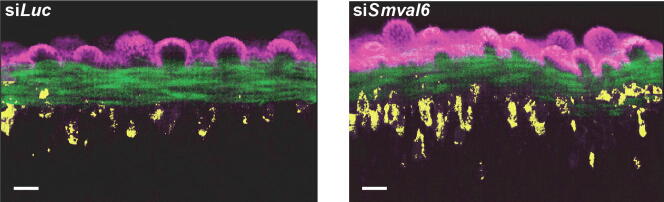

## 1 Introduction

Adult male and female schistosome pairs are master modulators of their environment (DeMarco et al., 2010, Wilson, 2012b, Robinson et al., 2013) and display developmental features evolutionarily honed for survival in one of the most inhospitable biological settings, definitive host mammalian blood. One particular anatomical adaptation that enables both schistosome sexes to maintain long-term, intravascular residence is the syncytial tegument; a structure covered by a unique plasma membrane architecture comprised of multiple stacks of lipid bilayers (McLaren and Hockley, 1977, Skelly and Wilson, 2006). Effectively, the schistosome’s tegument covers every host-interactive interface (body and blind ending gut) and triply functions as: (i) a barrier to host immunological and physiological defences, (ii) a dynamic layer for nutrient acquisition and (iii) a regulator of metabolic waste (Skelly and Wilson, 2006, Faghiri et al., 2010). Glycoprotein composition of this important parasite structure includes transmembrane candidates found on or between the surface membranes as well as embedded within tegumental organelles (Braschi et al., 2006, Braschi and Wilson, 2006, Mulvenna et al., 2010), glycosyl-phosphatidyl inositol (GPI) modified representatives (Koster and Strand, 1994, Castro-Borges et al., 2011) and numerous cytoplasmic constituents (van Balkom et al., 2005). The cytoplasm within this syncytial structure also contains mitochondria and two classes of secreted (from sub-tegumental cell bodies) inclusions termed discoid bodies and membranous bodies (Hockley, 1973). While the function of discoid bodies is unresolved (likely contributing to the maintenance of tegumental ground matter), the less numerous membranous bodies fuse with the apical tegumental membranes, contributing to their repair and maintenance (Wilson and Barnes, 1977).

Due to the key importance of maintaining normal tegumental functions during schistosome lifecycle progression, the (glyco) proteins contained within it have often been the subject of detailed functional characterisation (Da'dara et al., 2012) and/or immunoprophylactic-based investigations (Wilson, 2012a). One particular protein found enriched in tegumental extracts is *Schistosoma mansoni* venom allergen like protein 6 (SmVAL6) (van Balkom et al., 2005, Rofatto et al., 2012, Sotillo et al., 2015), an atypical member of a large schistosome protein family sharing sequence similarity to Sperm-Coating Protein/Tpx-1/Ag5/PR-1/Sc7 (SCP/TAPS) domain-containing representatives (Chalmers and Hoffmann, 2012). Previous studies have indicated that the gene encoding SmVAL6 is developmentally regulated, sex-associated (male > female) and alternatively spliced; the molecular processing is focused entirely on exons 3′ to those coding for the conserved SCP/TAPS domain (Chalmers et al., 2008, DeMarco et al., 2010, Rofatto et al., 2012). Interestingly, while some SmVAL6 isoforms have been linked to tegumental membranes, other variants are enriched in cytosolic fractions derived from the syncytium (Rofatto et al., 2012); at least one of these is also the target of human IgE responses (Farnell et al., 2015). Collectively, these findings have led to the supposition that SmVAL6 confers a yet to be identified adaptive advantage for adult schistosomes living in the definitive host vasculature. However, to date, a definitive role for any SmVAL6 isoform in schistosome tegumental (or wider biological) processes has yet to be revealed. Here, applying temporal and spatial gene expression analysis methods, loss-of-function RNA interference (RNAi) approaches and yeast 2-hybrid (Y2H) assays, we have conducted the first known investigation exploring SmVAL6 function in adult schistosomes.

## 2 Materials and methods

### 2.1 Ethics statement

All mouse procedures performed at Aberystwyth University (AU, United Kingdom) adhered to the United Kingdom Home Office Animals (Scientific Procedures) Act of 1986 (project licenses PPL 40/3700 and P3B8C46FD) as well as the European Union Animals Directive 2010/63/EU and were approved by AU Animal Welfare and Ethical Review Body (AWERB). In adherence to the Animal Welfare Act and the Public Health Service Policy on Humane Care and Use of Laboratory Animals, all mouse procedures performed at the University of Texas Southwestern Medical Center, USA, were approved by the Institutional Animal Care and Use Committee (IACUC) (protocol approval number APN 2017-102092).

### 2.2 Parasite material

A Puerto Rican strain (NMRI) of *S. mansoni* was used in this study. Mixed-sex worms were perfused from percutaneously infected TO (HsdOla:TO, Tuck-Ordinary, Envigo, UK) or Swiss-Webster (Charles River, USA) mice challenged 7 weeks earlier with 180 cercariae (Duvall and Dewitt, 1967) and used for RNAi, whole mount in situ hybridisation (WISH) and endpoint reverse transcription (RT)-PCR.

### 2.3 SmVAL6 transcription profile

Data from the 37,632 element *S. mansoni* long oligonucleotide DNA microarray studies of Fitzpatrick et al. (2009) was interrogated to find the expression profile of *Smval6* (Smp_124050) across 11 different lifecycle stages. Raw and normalised fluorescent intensity values are available via Array Express under the experimental accession number **E-MEXP-2094**.

### 2.4 Whole mount in situ hybridisation (WISH) of smval6

Adult worm fixation, processing and WISH were performed as previously described (Collins et al., 2013).

### 2.5 Single-cell RNA-Seq (scRNA-Seq) analysis

Localisation of *smp_095360* (*Sm14*), *smp_158660* (*dlc*) and *smp_124050* (*Smval6*) found within the 68 adult worm clusters generated from available scRNA-Seq data (Wendt et al., 2020) was depicted as uniform manifold approximation and projection (UMAP) plots using Seurat V3 (Stuart et al., 2019).

### 2.6 Short interfering RNA (siRNA)-mediated SmVAL6 silencing

Short interfering RNAs (siRNAs obtained from integrated DNA technologies (IDT), USA) were used to silence *Smval6* in both adult male and female schistosomes as previously described (Geyer et al., 2011; Geyer et al., 2018). siRNAs designed for firefly luciferase functioned as a negative control. All siRNA sequences used in this study are described in Supplementary Table S1. *Smval6* transcript abundance was measured by quantitative RT-PCR (qRT-PCR) at 48 h post siRNA treatment. Qualitative assessment of SmVAL6 protein abundance (western blot analysis) and quantification of adult worm phenotypes (laser scanning confocal microscopy, LSCM) was performed at day 7 post siRNA treatment.

### 2.7 Dextran staining of adult worms and LSCM

Adult male and female worms, untreated (*n* = 3 for each sex) or those treated with *luc* (*n* = 3 for each sex) or *Smval6* (*n* = 3 for each sex) siRNAs for 7 days were labelled with biotin-TAMRA-dextran reconstituted in either hypotonic (ultrapure water) or isotonic (DMEM) solutions for 10 min prior to fixation and labelling with Alexa-Fluor 488-conjugated phalloidin (Life Technologies) as previously described (Wendt et al., 2018). Post-labelling fixation of worms and mounting onto microscope slides were also performed as previously described (Wendt et al., 2018). LSCM images were acquired using a Leica SP8 confocal microscope equipped with a HC PL APO 63×/1.20 lens (Leica Microsystems, Germany), accruing a total of 50 sections for each Z-stack (step size of 0.365 µm). For each z-stack, the fluorescence intensity of the biotin-TAMRA-dextran channel was used to calculate the total volume (μm^3^) occupied by the fluorophore using the Surface tool in Imaris v8.2 (Bitplane). Volume measurements were taken from a 123 × 50 × 18 µm area located directly below the tegument. All voxels with an intensity over 10 arbitrary units (a.u.) or 15 a.u. were included for female and male worms, respectively.

### 2.8 Quantitative reverse transcription (qRT)-PCR and endpoint RT-PCR analyses

*Schistosoma mansoni* total RNA isolation and qRT–PCR analyses were performed as previously described (Chalmers et al., 2008, Geyer et al., 2011). A StepOnePlus thermocycler (Applied Biosystems) was used for all qRT-PCR assays with *Smval6* gene expression results normalised to *α-tubulin* (Smp_090120). Endpoint RT-PCR was performed using adult worm cDNA essentially as described (Fitzpatrick et al., 2008). Dideoxy chain termination DNA sequencing of endpoint PCR products was performed at the Institute of Biological, Environmental and Rural Sciences (IBERS) (Aberystwyth University, United Kingdom) translational genomics facilities. qRT–PCR and endpoint RT-PCR primer sequences for *Smval6*, *α-tubulin, Sm14* and *Sm14delta e3* amplicons are given in Supplementary Table S1.

### 2.9 Yeast assays

Truncated versions of SmVAL6 (Smp_124050) were sub-cloned into the Y2H GAL4 DNA-BD fusion vector pGBKT7 and sequence verified. Each pGBKT7-SmVAL ‘bait’ construct was introduced into yeast strain Y187 using the lithium acetate transformation protocol (Gietz et al., 1992) and tested for auto-activation, toxicity and expression as described in the Matchmaker™ Library Construction and Screening Kit manual (Clontech). Total protein extracts from transformed yeast cells were obtained using urea/SDS, phenylmethylsulfonylfluoride (PMSF, Sigma-Aldrich) and a protease inhibitor cocktail tablet (Complete Mini, Roche) as described in the yeast protocols handbook (Clontech). Protein extracts were subsequently analysed for SmVAL6 expression by standard SDS-PAGE and western blotting.

Mating reactions were performed between the haploid pGBKT7-SmVAL6 Y187 yeast transformants and a 7 week mixed-sex adult worm pGADT7-‘prey’ library transformed in AH109 (donated by Professor Alex Loukas, James Cook University, Australia), plated onto triple dropout synthetic medium (TDO medium, SD/–Trp/–Leu/–His) and incubated at 30 °C for 4–7 days as previously described (Geyer et al., 2018). Replica patches of all colonies were streaked onto TDO medium and quadruple dropout medium (QDO medium, SD/–Trp/–Leu/–His/–Ade), and incubated for a further 4 days to confirm activation of the ADE2 reporter. Activation of the MEL1 reporter was assayed colorimetrically on QDO containing X-alpha-GAL. LACZ reporter activity of all positive colonies was then tested using colony-lift assays as described in the Yeast protocols handbook (Clontech).

Prey plasmids of interest were rescued from yeast using the Easy Yeast Plasmid Isolation kit (Clontech) and propagated in α-select *Escherichia coli* cells (Bioline). Prey clones were sequenced using either the 5′ or 3′ long distance (LD) amplimer described in the Matchmaker™ Library Construction and Screening Kit (Clontech). Sequences were queried against the reference *S. mansoni* genome (v7.0) using BLAST. All schistosome open reading frames (ORFs) were checked to ensure that they were in frame with the GAL4 activation domain (GAL4-AD), thereby ensuring correct expression of the fusion proteins in yeast.

To confirm protein–protein interactions, a representative of each identified prey (Sm14 DeltaE3/Smp_095360.2; dynein light chain/Smp_158660) and its respective bait (SmVAL6T2, SmVAL6T1) were co-transformed into the Y2HGold strain (Gietz et al., 1992). Full-length Sm14 (Sm14FL/Smp_095360) was also co-transformed with SmVAL6T1 into the Y2HGold strain. p53+SV40 Large T-antigen and lamin C+SV40 Large T-antigen bait-prey combinations supplied with the Clontech kit were used as positive and negative controls, respectively. To test for prey auto-activation, the empty pGBKT7 vector was co-transformed with each isolated prey library construct. Co-transformants were selected for on SD-Trp/-Leu (DDO, double dropout medium) following incubation at 30 °C for 3–4 days. Growing colonies were then replica streaked onto DDO, QDO/+X-α-GAL and QDO/+X-α-GAL/+Aureobasidin A selection media to confirm protein–protein interactions in yeast.

Quantitative β-galactosidase assays were carried out to assess the relative strength of protein–protein interactions. *Ortho*-Nitrophenyl-β-galactoside (ONPG) assays were carried out as described in Clontech’s Yeast Protocols Handbook. Pellet X-β-gal (PXG) assays were carried out as previously described (Mockli and Auerbach, 2004).

### 2.10 SDS-PAGE and western blotting

For total parasite protein extraction, 7-week male worms were homogenised in lysis buffer (20 mM KH_2_PO_4_, pH 7.4 and 0.1% v/v Triton X-100) using a Tissue Lyser (Qiagen). Soluble yeast proteins (described in section 2.9) and parasite extracts were electrophoresed on NuPAGE Novex pre-cast 4–12% Bis-Tris gradient gels (Invitrogen) and transferred onto polyvinylidene fluoride (PVDF) membranes in NuPAGE transfer buffer (Invitrogen). Membranes were blocked in PBS/Tween-20 (0.3% v/v) containing 5% skimmed milk powder (blocking buffer) overnight at 4 °C before incubation with antibodies. In the case of yeast blots, a GAL4-BD-horseradish peroxidase (HRP)-linked antibody (Santa Cruz Biotechnology) was used (1:1000 dilution for 1 h) in PBS/Tween-20 (0.3% v/v) containing 1% skimmed milk powder. In the case of parasite blots, the membrane was incubated with anti-rSmVAL6 (1:600 dilution in blocking buffer) for 3 h (Rofatto et al., 2012). Following 3 × 10 min washes in PBS/Tween-20 (0.3% v/v), the membrane was incubated with goat anti-mouse IgG conjugated HRP (1:2000 in blocking buffer) for 1 h. All blots received a final 3× wash in PBS/Tween-20 (0.3% v/v) and were subsequently developed using ECL-Plus reagent (GE Healthcare).

### 2.11 Structural modelling of SmVAL6 complexes

The structural model of a truncated form of Smp_124050/SmVAL6 (Uniprot (UniProt, 2009) ID. Q1XAN2) and Smp_158660/DLC (Uniprot (UniProt, 2009) ID. G4LZ86) were derived by homology modelling using M4T (Fernandez-Fuentes et al., 2007). The crystal structure of GAPR-1 (PDB (Berman et al., 2000) code 1smb ((Serrano et al., 2004)) and DLC TcTex-1 (PDB code 1ygt (Williams et al., 2005)) were used as templates to model SmVAL6 and DLC, respectively. The expected value (E-value) and the percentage of conserved residues (shown in parentheses) of GAPR-1 and SmVAL6 and TcTex-1 and DLC are 2e^−21^ (60%) and 1e^−10^ (55%), respectively, with sequence coverage above 90% in both cases. The quality and stereochemistry of the models were assessed using ProSA-II (Sippl, 1993) and PROCHECK (Laskowski et al., 1993), respectively. The structural modelling of protein complexes was done using rigid body docking. The structural models of SmVAL6 and DLC as described above and the crystal structure of Smp_095360/Sm14 (PDB code: 1vyg (Angelucci et al., 2004) were used as inputs to derive models for the binary complexes SmVAL6-DLC and SmVAL6-Sm14. The docking space was sampled using ZDOCK 3.02 (Mintseris et al., 2007), generating 10, 000 docking poses for each of the complexes. The docking complexes were then ranked using pyDOCK (Cheng et al., 2007). The top 100 docking poses were used to compute the preferred SmVAL6 interface patches for both Smp_158660/DLC and Smp_095360/Sm14.

## 3 Results

### 3.1 Smval6 temporal and spatial expression

**Fig. 1:**
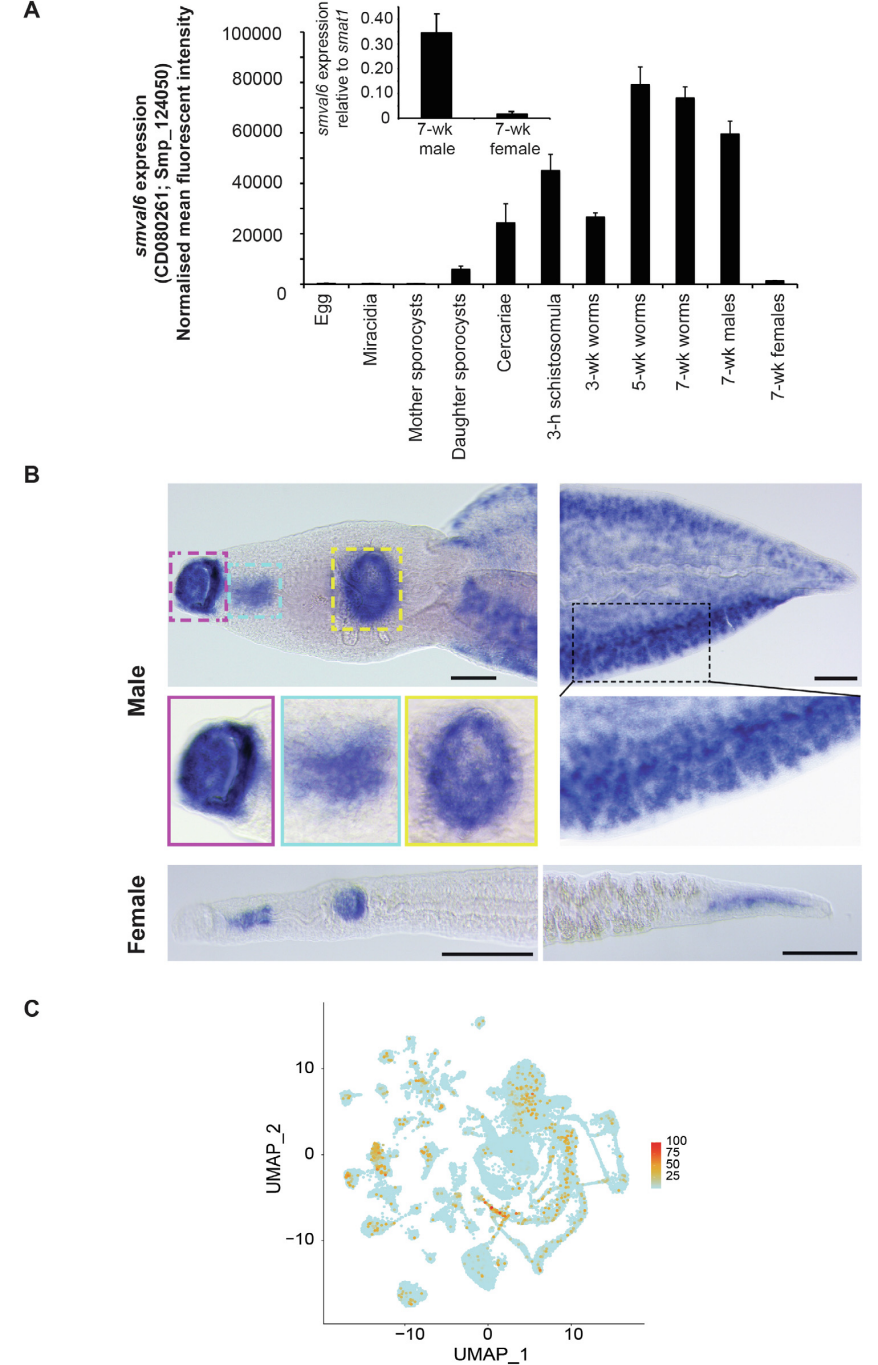
Developmentally-regulated *Schistosoma mansoni* venom allergen-like gene *Smval6* is expressed in the oral sucker, acetabulum, anterior region of the oesophageal gland and mesenchymal cells of adult worms. (A) DNA microarray analysis of *Smval6* (*smp_124050*) expression throughout 11 lifecycle stages. Bar chart represents normalised mean fluorescent intensities + S.D. (*n* = 3 replicates/lifecycle stage except adult female, where *n* = 2) of *Smval6* transcript abundance derived from oligonucleotide CD080261 as described previously (Fitzpatrick et al., 2009). Inset box represents quantitative reverse transcription-PCR (qRT-PCR) analysis of *Smval6* expression in adult male and female schistosomes. qRT-PCR primer sequences are included in Supplementary Table S1. (B) Expression of *smval6* in adult somatic tissues reveals localisation (blue signal) to the oral sucker, anterior region of the oesophageal gland (AOG), acetabulum and cells scattered through the mesenchyme. Scale bars, 100 μm. Where illustrated, delineated areas (dashed boxes) are magnified in the insets (solid boxes). Magenta, oral sucker; cyan, AOG; yellow, acetabulum; black, mesenchyme/sub-tegumental cells. Due to comparably low (compared with males) abundance, *Smval6* expression was more difficult to detect in adult female tissues (with lack of detectable signal in the oral sucker represented in this individual). (C) Uniform manifold approximation and projection (UMAP) plot of *smp_124050* (*Smval6*) found within the 68 adult worm clusters generated from available single cell (sc)RNA-Seq data (Wendt et al., 2020).

**Supplementary figure 1:**
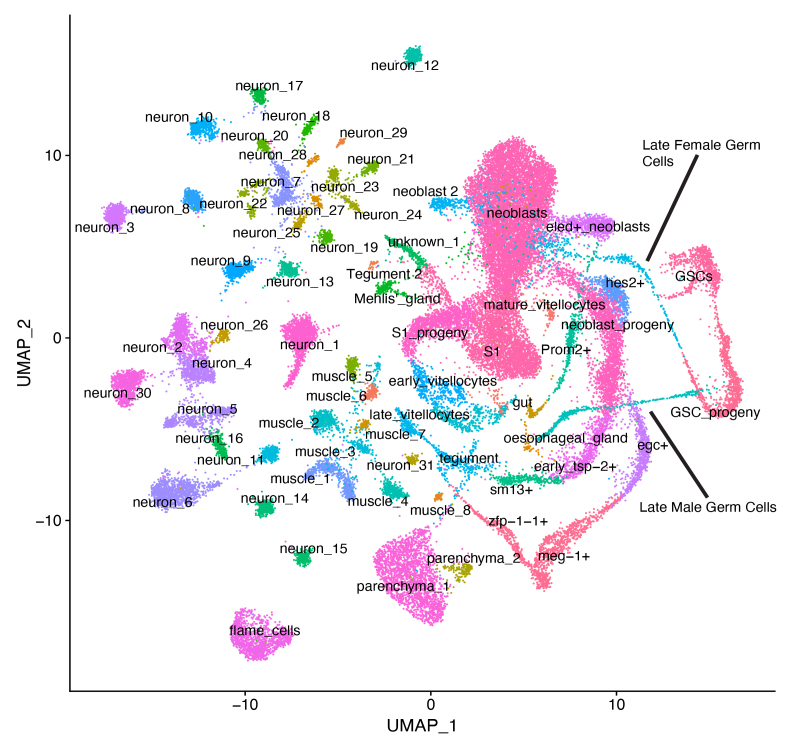
Uniform manifold approximation and projection (UMAP) view of all identifiable cellular clusters in adult schistosomes. The 68 clusters identified by Wendt et al. (2020) are projected here with labels. Cells are derived from sexually-mature adult male and female worms as well as sexually immature (developed in the absence of males) females. **Reference:** Wendt, G., Zhao, L., Chen, R., Liu, C., O’Donoghue, A.J., Caffrey, C.R., Collins, J.J., 2020. A single-cell RNAseq atlas of the pathogenic stage of *Schistosoma mansoni* identifies a key regulator of blood feeding. Science 369 (6511), 1644-1649.

While our earlier studies revealed developmentally regulated and gender-associated *Smval6* expression in schistosomes (Chalmers et al., 2008, Rofatto et al., 2012) (confirmed here in Fig. 1A by both qRT-PCR and DNA microarray analyses), spatial expression of this parasite gene product in those investigations was not thoroughly described. Therefore, to reveal where *Smval6* expression was found throughout adult schistosome tissues, we conducted WISH assays in both male and female parasites (Fig. 1B). Broadly supporting previous studies (Rofatto et al., 2012, Fernandes et al., 2017), adult male *Smval6* expression was found to be concentrated in both oral (Fig. 1B, red box) and ventral (Fig. 1B, yellow box) suckers. However, our results also demonstrated wide distribution of *Smval6* expression throughout adult male mesenchymal tissues (Fig. 1B, black box) as well as to the anterior region (Fig. 1B, cyan box) of the oesophageal gland (AOG (Li et al., 2013)). A similar pattern was also observed in female schistosomes. However, as females express *Smval6* at much lower levels compared with males (Fig. 1A), the mesenchymal-, ventral sucker and ACM signals were much weaker in this sex. In fact, in this individual female, *Smval6* expression was completely absent in the oral sucker. To further define which tissues throughout the mesenchyme were expressing *Smval6*, we consulted a single cell RNA-seq (scRNA-Seq) atlas of adult schistosomes (Wendt et al., 2020). Although *Smval6* expression was found in a variety of mesenchymal tissues (e.g. neurons, flame cells and neoblasts), it was particularly enriched in tegumental cell bodies (Fig. 1C and Supplementary Fig. S1).

### 3.2 Smval6 siRNA-mediated knockdown

**Fig. 2:**
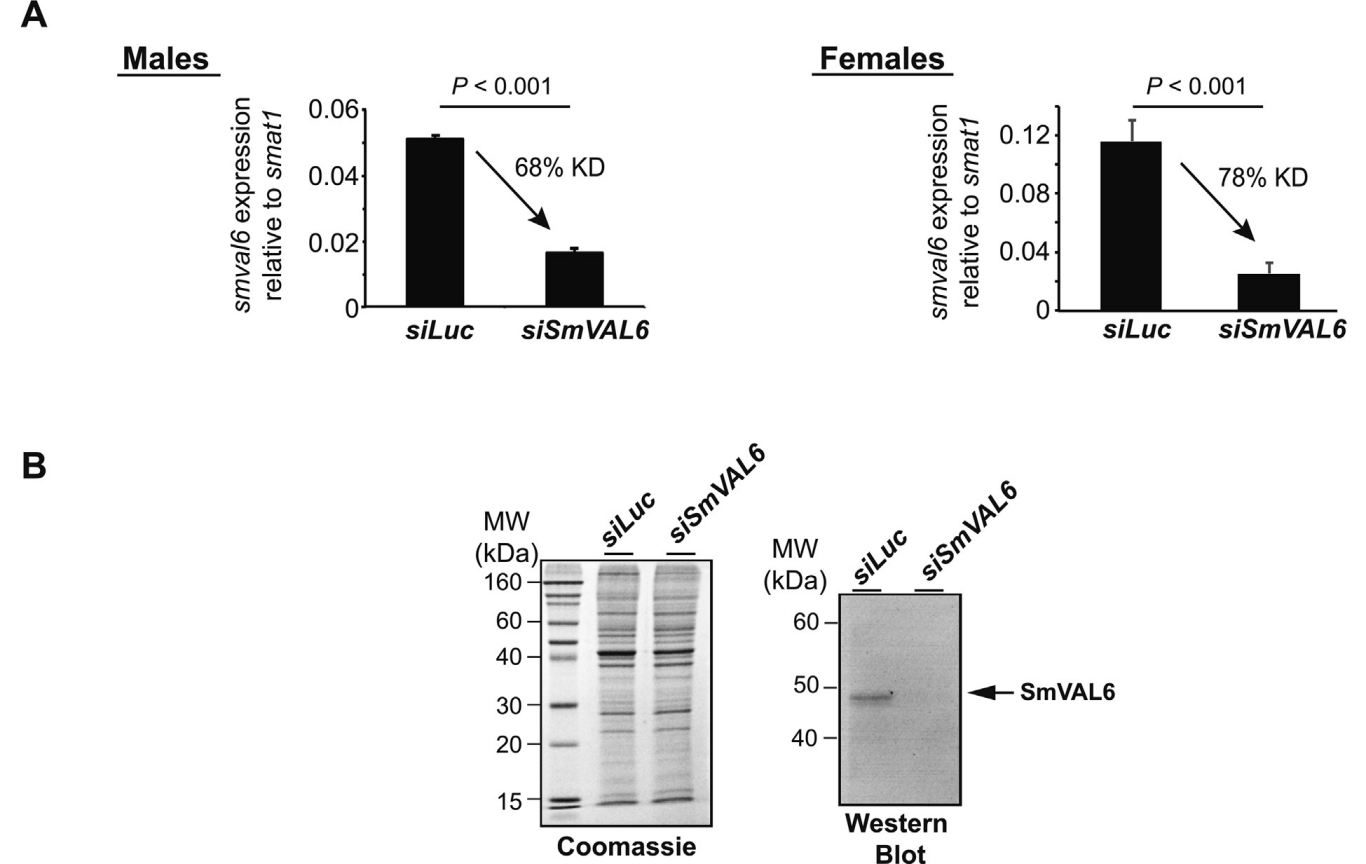
*Schistosoma mansoni* venom allergen-like gene *Smval6*-mediated RNA interference (RNAi) of adult worms depletes both transcript and protein levels. (A) Seven week old adult male and female schistosomes were electroporated with 5 µg of short interfering RNA (siRNA) duplexes targeting either *luciferase* (si*Luc*) or *Smval6* (si*Smval6*). After 48 h, total RNA was harvested and subjected to quantitative reverse transcription (qRT)-PCR. Percent knockdown (KD) is indicated. All siRNA and qRT-PCR DNA sequences are included in Supplementary Table S1. (B) After 7 days, total protein was harvested and subjected to SDS-PAGE and western blot analyses using a murine polyclonal anti-rSmVAL6 antisera (Rofatto et al., 2012).

**Fig. 3:**
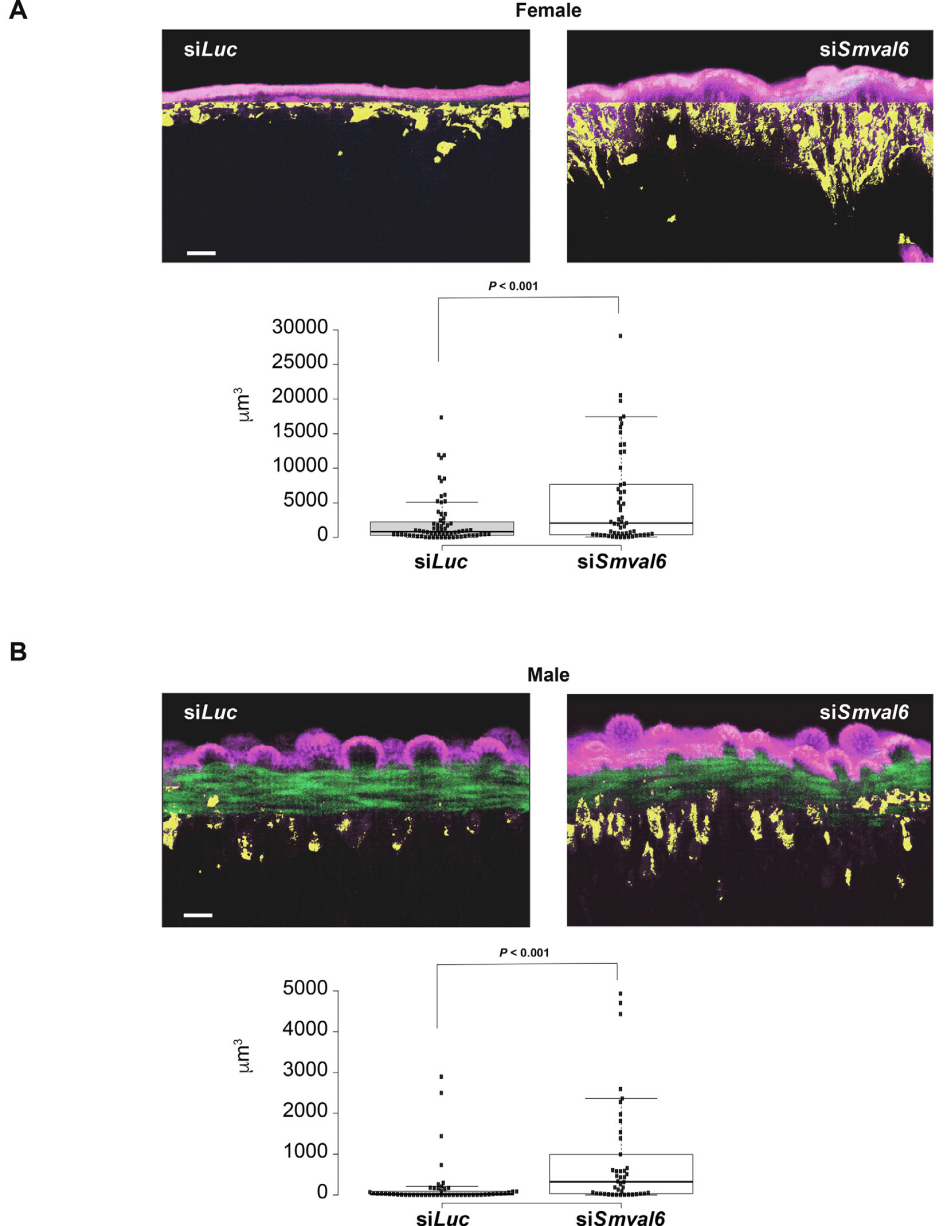
*Schistosoma mansoni* venom allergen-like protein SmVAL6 regulates tegumental barrier function in adult schistosomes. Seven week old male and female schistosomes were electroporated with either short interfering (si)*Smval6* or si*Luciferase* (si*Luc*) duplexes (as described in section 2 and Fig. 2 legend). At 7 days, the worms were labelled with biotin-TAMRA-dextran, fixed, labelled with Alexa-Fluor 488-conjugated phalloidin and mounted onto microscope slides as previously described (Wendt et al., 2018). Mounted worms were then subjected to LSCM as described in the section 2. (A) Representative view of adult female worms (si*Luc*, *n* = 3; si*Smval6*, *n* = 3) together with a box and whisker chart of all collected data (si*Luc* sections from three worms, *n* = 67; si*Smval6* sections from three worms, *n* = 55). (B) Representative view of adult male worms (si*Luc, n* = 3; si*Smval6*, *n* = 3) together with a box and whisker chart of all collected data (si*Luc* sections from three worms, *n* = 51; si*Smval6* sections from three worms, *n* = 42). Alexa-fluor 488-conjugated phalloidin, green; biotin-TAMRA-dextran, pink (outside) and yellow (inside). Scale bars = 10 µm.

Whereas functions for distantly related SmVALs have been linked to lipid binding (SmVAL4 (Kelleher et al., 2014)), extracellular matrix remodelling (SmVAL9 (Yoshino et al., 2014)) and plasminogen binding (SmVAL18 (Fernandes et al., 2018)), a role for SmVAL6 in any aspect of schistosome biology or host interactions has yet to be determined. Therefore, to assess the significance of *Smval6* loss-of-function in both adult male and female schistosomes (lifecycle stages where *Smval6* localisation is known, Fig. 1B and C), siRNA-mediated knockdown was employed (Fig. 2). Here, RNAi was reproducibly efficient in suppressing *Smval6* transcript levels in both sexes (68% in males, 78% in females) compared with control worms (*siLuc* treated) as quantified by qRT-PCR (Fig. 2A). This *siSmval6*-mediated reduction in transcript levels also correlated with measurable decreases in protein abundance as determined by western blot analyses (using a polyclonal antisera raised against recombinant SmVAL6, (Rofatto et al., 2012)) of soluble male worm extracts (Fig. 2B). We were unable to detect SmVAL6 in soluble female extracts (regardless of the siRNAs used), presumably due to the low abundance of this protein ((Rofatto et al., 2012) and inferred from Fig. 1A). Consistent with enriched expression of *Smval6* in the parasite tegumental cell bodies, we noted that surface membranes of *siSmval6*-treated parasites were noticeably affected in comparison to si*Luc* controls (Supplementary Fig. S2) and suggested a SmVAL6-regulated phenotype (membrane integrity). Therefore, to objectively quantify surface membrane integrity differences between si*Luc-* and si*SmVal6*-treated worms, a recently described method for fluorescently labelling (using biotin-TAMRA-dextran) the tegument and sub-tegumental projections/cell bodies was utilised (Wendt et al., 2018) (Fig. 3).

Here, using this live worm labelling technique, a clear and significant increase in dextran permeability through the tegument and into the sub-tegumental cell bodies of si*Smval6*-treated adult female (Fig. 3A) and male (Fig. 3B) worms was observed compared with control si*Luc*-treated parasites. While this increase in surface permeability was not as dramatic as that seen in adult schistosomes exposed to hypotonic conditions (Supplementary Fig. S3), these results clearly illustrated the importance of SmVAL6 in mediating tegumental integrity.

### 3.3 SmVAL6 interacting partners

**Fig. 4:**
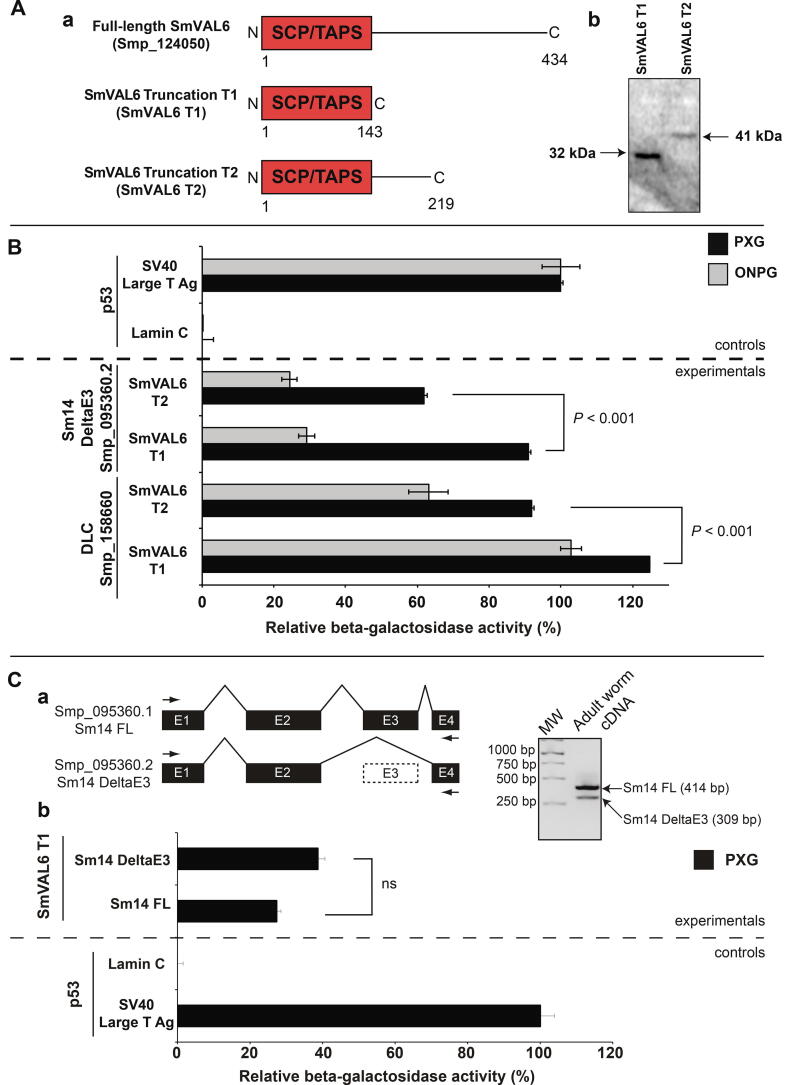
*Schistosoma mansoni* venom allergen-like protein SmVAL6 interacts with both a fatty acid binding protein (Sm14) and a dynein light chain (DLC). (A) Sperm-Coating Protein/Tpx-1/Ag5/PR-1/Sc7 Protein/Tpx-1/Ag5/PR-1/Sc7 (SCP/TAPS)-containing SmVAL6 truncations T1 (143 aa; 32 kDa) and T2 (219 aa; 41 kDa) were generated from full-length SmVAL6 (Smp_124050) (a) and expressed in *Saccharomyces] cerevisiae* to identify adult worm interacting partners during yeast-2-hybrid (Y2H) screens (b). (B) The strength of SmVAL6/Sm14 DeltaE3 (Smp_095360.2) and SmVAL6/DLC (Smp_158660) interactions was quantified using both the pellet X-β-gal (PXG; black bars) (Mockli and Auerbach, 2004) and the *Ortho*-Nitrophenyl-β-galactoside (ONPG; grey bars) assays. Student’s *t*-tests were used to detect the significance of differential interactions quantified by the more sensitive PXG assay (SmVAL6T1/Sm14 DeltaE3 versus SmVAL6T2/Sm14 DeltaE3; SmVAL6T1/DLC versus SmVAL6T2/DLC). (C) Adult worms express alternatively spliced versions of Sm14: full-length Sm14 (Sm14 FL, Smp_095360.1) and the alternatively spliced Sm14 lacking exon 3 (Sm14 DeltaE3, Smp_095360.2). Gene structures are depicted where ‘E’ represents exons and arrows indicate PCR primers used to amplify both Sm14 isoforms (a). SCP/TAPS-containing SmVAL6 T1 interacts with both Sm14 isoforms as quantified by the PXG assay (b). ONPG and PXG controls included p53 + SV40 large T antigen (positive) and p53 + Lamin C (negative).

Identifying SmVAL6 interacting proteins or complexes may help further define the role of this particular SmVAL, but more importantly, may provide an explanation for the surface membrane damage observed in si*SmVAL6*-treated adult worms. To this end, we performed Y2H screens of adult schistosome cDNA libraries to search for potential SmVAL6 protein interactors (Fig. 4). Two different SmVAL6 constructs were created for these Y2H screens; one (SmVAL6 truncation 1, SmVAL6 T1) contained only the SCP/TAPS domain whereas a second one (SmVAL6 truncation 2, SmVAL6 T2) contained the SCP/TAPS domain including a C-terminal 73 amino acid extension encoded by the full-length gene (Fig. 4Aa). Upon transfection into yeast cells, both SmVAL6 T1 and T2 constructs expressed proteins of the predicted molecular masses (Fig. 4Ab). Analyses of the Y2H screens indicated that both SmVAL6 T1 and T2 interacted with a previously characterised fatty acid binding protein isoform (C-terminal 68 aa of Sm14 DeltaE3 (Ramos et al., 2003), Smp_095360.2) as well as a DLC (C-terminal 110 aa of DLC, Smp_158660). The relative interactive strengths of SmVAL6 and its binding partners were investigated using two different quantitative assays (ortho-Nitrophenyl-β-galactoside, ONPG; pellet X-β-galactoside, PXG) and illustrated that SmVAL6 T1 had stronger affinities (*P* < 0.001; using the more sensitive PXG assay) for both Sm14 DeltaE3 and DLC than did SmVAL6 T2 (Fig. 4B).

Because the full-length, fatty acid binding protein Sm14 (Sm14 FL, Smp_095360.1 (Moser et al., 1991)) was not detected in the original SmVAL6 Y2H screens, we next investigated whether the intact variant could interact with SmVAL6 (Fig. 4C). Endpoint RT-PCR, using PCR primers capable of amplifying both Sm14 FL and Sm14 DeltaE3 (Supplementary Table 1), clearly demonstrated the presence of these two fatty acid binding variants in adult worm cDNA samples (Sm14 FL > Sm14 DeltaE3, Fig. 4Ca); these transcription results broadly confirmed the findings of Ramos et al. (2003). Subsequent SmVAL6-Sm14FL protein–protein interactions (PPi) were investigated using the more sensitive (compared with ONPG, Fig. 4B) quantitative PXG assay (Mockli and Auerbach, 2004) (Fig. 4Cb). Here, we focused on SmVAL6 T1 for these PPi assays due to the greater affinity of this variant for the targets identified (Fig. 4B). Despite not being detected in the original Y2H screens (possibly related to low abundance of this variant in the Y2H library), PXG assays demonstrated that Sm14 FL was, indeed, capable of binding to SmVAL6. However, the Sm14 FL/SmVAL6 interaction strength was slightly less than that detected for Sm14 DeltaE3/SmVAL6 interactions (not significant).

### 3.4 SmVAL6/Sm14 FL/DLC are co-expressed and are likely to form higher order protein complexes in adults

**Fig. 5:**
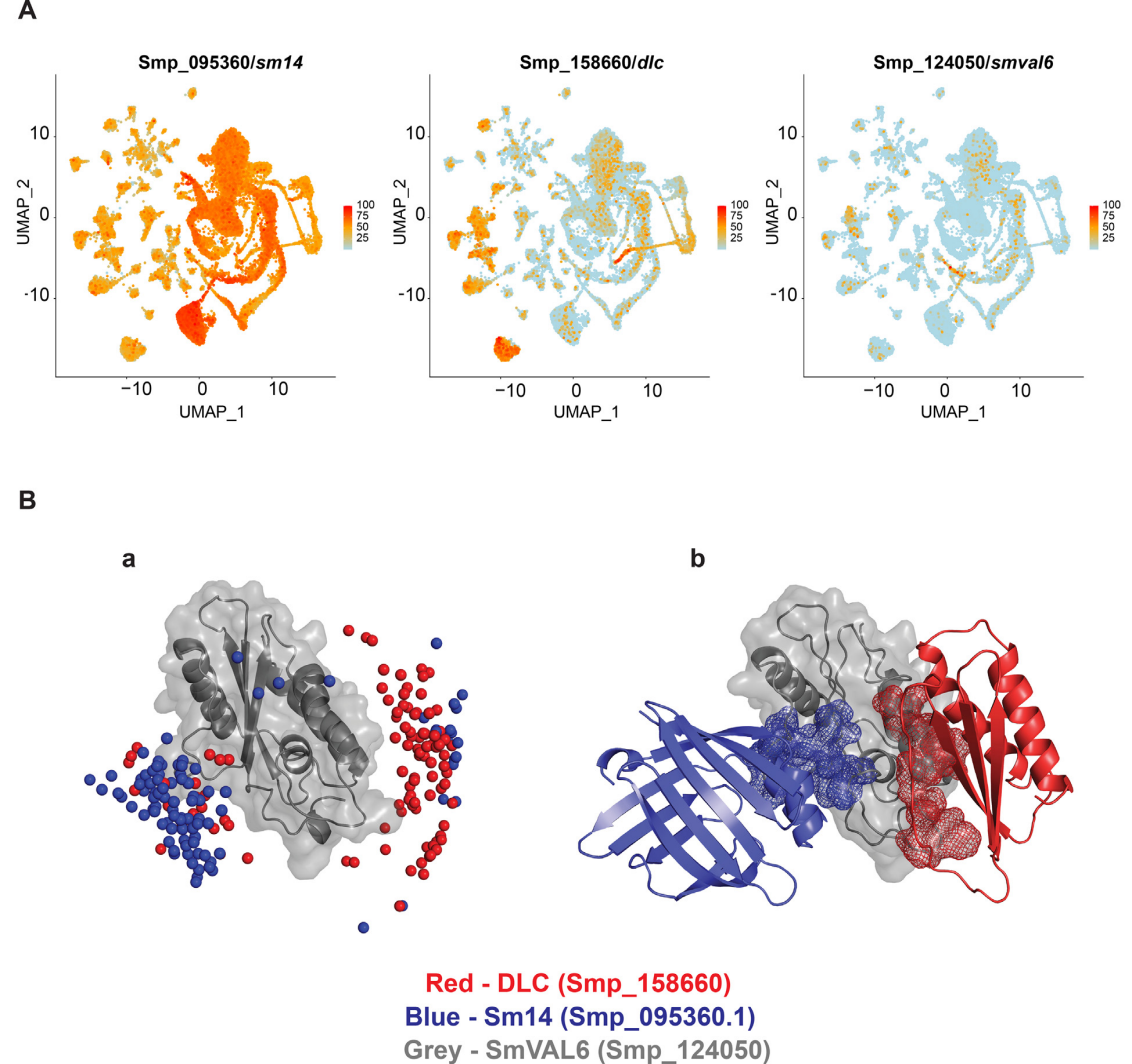
Single-cell RNA sequencing (scRNA-Seq)-facilitated localisation of transcripts and structural homology modelling of protein complexes supports *Schistosoma mansoni* venom allergen-like protein SmVAL6/Sm14 and SmVAL6/dynein light chain (DLC) interactions. (A) Uniform manifold approximation and projection (UMAP) plots of *smp_095360* (*sm14*), *smp_158660* (*dlc*) and *smp_124050* (*Smval6*) found within the 68 adult worm clusters generated from available scRNA-Seq data (Wendt et al., 2020). (B) Binary SmVAL6-DLC and SmVAL6-Sm14 complexes were created as described in section 2. The top 100 docking poses (a) were used to compute the preferred SmVAL6 interface patches for both Smp_158660/DLC (red spheres) and Smp_095360/Sm14 (blue spheres). The average preferred SmVAL6 interacting patch (b) is also indicated for DLC (red) and Sm14 (blue); notice how these occupy opposite faces of SmVAL6.

Interrogation of available scRNA-Seq data was used to provide evidence in support of the SmVAL6/Sm14 FL and SmVAL6/DLC PPis identified in the Y2H assays (Fig. 5A). In adult schistosomes, *Sm14* expression was found disseminated throughout most cell types including the mesenchyme/parenchyme clusters Supplementary Fig. S3; this localisation broadly supported the Sm14 protein distribution reported by both Moser et al., 1991, Gobert, 1998 in *S. mansoni* and *S. japonicum*, respectively. While *dlc* was also found broadly distributed throughout adult schistosome tissues, it was less abundant than *Sm14* (with the exception of flame cells and neurons)*.* Importantly, clusters of *Smval6+* cells (e.g. neurons, tegumental cells, neoblasts) were also found to be co-expressing both *dlc* and *Sm14* (Fig. 5A and Supplementary Fig. S1).

The scRNA-seq data indicated that *Smval6*, *Sm14* and *dlc* were potentially co-expressed in a small proportion of cells throughout adult tissues. As the Y2H results also demonstrated that SmVAL6 T1 formed specific and direct interactions with both Sm14 FL and DLC (Fig. 4), we initiated molecular modelling with the three schistosome proteins to understand how these molecular complexes could be formed (Fig. 5B). Specifically, we used the SCP/TAPS region of SmVAL6 (SmVAL6 T1 only contained the SCP/TAPS domain; Fig. 4A) as well as full-length Sm14 and DLC to construct the models. Examining the top 100 (out of 10, 000) docking poses illustrated that two distinct SmVAL6 interfaces were predominantly used for Sm14 and DLC interactions (Fig. 5Ba). While some overlap between Sm14 and DLC interfaces existed in the predicted models, the dominant SmVAL6 interacting interface derived from averaging the top 100 docking poses for Sm14 and for DLC were on opposite faces of the SCP/TAPS domain (Fig. 5Bb).

## 4 Discussion

The SmVAL family contains both excreted/secreted (SmVAL group 1) as well as non-secreted (SmVAL group 2) members (Chalmers and Hoffmann, 2012). Despite temporal and spatial investigations revealing developmental (Chalmers et al., 2008) and tissue-associated patterns (Rofatto et al., 2012, Fernandes et al., 2017, Farias et al., 2019), how these proteins participate in parasite biology or host interactions remains largely enigmatic. Where functional studies have been performed, these have been restricted to the group 1 SmVALs and have indicated roles in host extracellular matrix reorganisation (SmVAL9) (Yoshino et al., 2014) as well as in plasminogen (SmVAL18 (Fernandes et al., 2018)) and lipid (SmVAL4 (Kelleher et al., 2014)) binding. Due to a dearth of information related to the biology of group 2 SmVALs, we present the first functional investigation of a representative family member, SmVAL6.

In addition to confirming previous studies describing the spatial distribution of *Smval6* to the oral and ventral suckers as well as the tegumental cells of adult schistosomes (Rofatto et al., 2012, Fernandes et al., 2017), we additionally localise this transcript to the AOG in both sexes (Fig. 1). Intriguingly, another group 2 SmVAL (*Smval13*) localises to the AOG; this contrasts with the localisation of *Smval7* (a group 1 SmVAL), which is enriched in the posterior region of the oesophageal gland (POG) (Fernandes et al., 2017). The only other transcripts localised (thus far) to the adult schistosome AOG are the microexon genes (MEGs) 12, 16 and 17 as well as phospholipase A2, while a total of 11 MEGs, two lysosomal hydrolases and one glycosyltransferase are localised to the POG (DeMarco et al., 2010, Li et al., 2013, Wilson et al., 2015). While the oesophageal gland contributes to erythrocyte/leukocyte lysing and digestion, the specific roles of SmVAL7, SmVAL13 and now SmVAL6 in this process are currently unknown. However, as the AOG has been postulated to be a holding area for cells during schistosome feeding in preparation for transport into the POG where cellular lysis occurs (Li et al., 2013), perhaps SmVALs differentially (excreted/secreted group 1 SmVALs contributing to lysis and non-secreted group 2 SmVALs contributing to maintaining structural characteristics for receiving a cellular bolus) participate in this critical process. Nevertheless, WISH/scRNA-Seq localisation of *Smval6* to the adult worm AOG, suckers and tegumental/mesenchymal cells in the current study, combined with the proteomic characterisation of SmVAL6 to adult worm tegumental fractions (van Balkom et al., 2005, Rofatto et al., 2012), suggests that this group 2 SmVAL may have more than one function. To help shed light on this subject, si*Smval6-*mediated RNAi of adult worms and Y2H screening for SmVAL6 interactors were subsequently performed.

The most striking phenotype observed in si*Smval6*-treated adult male and female worms was an increased distribution of biotin-TAMRA-dextran across the tegument and into the sub-tegumental cell bodies below the muscle layer (Fig. 3). This result indicates that SmVAL6 directly or indirectly participates in tegumental barrier function, in addition to oesophageal gland activities, in adult schistosomes. Two previous proteomics investigations identifying SmVAL6 in tegumental fractions (van Balkom et al., 2005, Rofatto et al., 2012), coupled with our WISH/scRNA-Seq localisation of *Smval6* to sub-tegumental (amongst other) cell bodies (Fig. 1B), supports this assertion by providing complementary spatial contexts for the role of SmVAL6 in tegumental biology. Within the tegument or sub-tegumental cell bodies, SmVAL6 (one or more of its alternatively spliced isoforms) may associate with cytoplasmic/organelle/surface membranes due to its predicted palmitoylation post-translational modifications (Rofatto et al., 2012). Interactions with organelle membranes have previously been described for golgi-associated PR-1 protein (GAPR-1) (Eberle et al., 2002), a cytoplasmic human homolog of SmVAL6. While this interaction is facilitated by GAPR-1 myristoylation (a related protein lipidation), it also is dependent on interactions with caveolin-1 (Eberle et al., 2002). Further studies of GAPR-1 have demonstrated that this protein can also self-assemble into oligomeric fibrils (Eberle et al., 2002, Olrichs et al., 2014) and can interact with both beclin-1, a potent inducer of autophagy (Shoji-Kawata et al., 2013) as well as TMED7, a TRAM-TRIF signalling pathway inhibitor (Zhou et al., 2016). When taken together, group 2 SCP/TAPs domain containing proteins such as SmVAL6 and GAPR-1 contain features that stabilise protein/lipid (perhaps similar to the group 1 SmVAL4 (Kelleher et al., 2014)) or protein/protein interactions. The surface membrane disruption phenotype induced by *Smval6* RNAi (Fig. 3), as well as the interaction of SmVAL6 with both the fatty acid binding protein Sm14 (FL and Delta E3 variants; Smp_095360.1 and Smp_095360.2, respectively) and a DLC (Smp_158660) (Fig. 4), mutually support this contention. However, it is currently unknown whether the marginal differential interaction strengths found for SmVAL6 with Sm14delta E3 > Sm14 variants (Fig. 4) affects the competitive regulation of lipid transfer or membrane turnover within schistosomes. This, together with identifying how SmVAL6 mediates these particular or additional molecular interactions, requires more thorough investigation.

As an initial step in addressing how SmVAL6 mediates molecular interactions of the proteins identified in this study, we undertook two different approaches. In the first approach, scRNA-seq localisation of *Smval6*, *Sm14* and *dlc* transcripts revealed the co-expression of all three transcripts to a small population of adult schistosome cells (i.e. tegumental cells, neurons and neoblasts) (Fig. 5A). These results provide in situ support for the Y2H findings and imply that SmVAL6-Sm14 and SmVAL6-DLC interactions could occur within adult schistosomes due to their spatial co-expression/co-localisation. For SmVAL6 and Sm14, additional experimental support for spatial localisation of these two proteins was recently provided by the analysis of adult worm extracellular vesicles (EVs). In this previous investigation, the 15K EV pellet contained both SmVAL6 and Sm14 in sufficient quantities to be detected by the LC-MS/MS methodologies employed (Kifle et al., 2020). Interestingly, these two proteins were found within the peripheral membrane proteomes of the 15K EV pellet, indicating their association with lipid-rich compartments and further supporting a role for SmVAL6 in maintaining membrane integrity. An in silico modelling approach was subsequently used to predict how SmVAL6 mediated these distinct protein–protein interactions. Here, we found that opposing faces of the SCP/TAPS region of SmVAL6 were differentially used to drive interactions with Sm14 or DLC (Fig. 5B). This finding agrees with the supposition that parasite (and possibly other metazoan) SCP/TAPS domains may operate as a flexible tertiary structure critical to roles (known and unknown) in diverse functional contexts (Hewitson et al., 2011). With regard to schistosome SmVALs, considering how opposing SCP/TAPS faces orchestrate diverse aspects of host interactions (predominantly Group 1 SmVALs) and parasite-specific activities (predominantly Group 2 SmVALs) should contribute to an increased functional understanding of this enigmatic platyhelminth protein family (Chalmers and Hoffmann, 2012).

Taken together, we provide direct evidence that SmVAL6 is necessary for maintaining barrier function of the tegument, likely through its interactions with both lipid and protein membrane constituents. Further roles in oesophageal function are implied, demonstrating that this Group 2 SmVAL may participate in diverse functions critical to schistosome biology.

## Appendix A Supplementary data

Supplementary Table S1

Supplementary figure S2 *Schistosoma mansoni* venom allergen-like protein SmVAL6 reductions impair tegumental barrier function in adult schistosomes. Seven week old female schistosomes were electroporated with either short interfering (si)*Smval6* (*n* = 16) or si*Luciferase* (si*Luc*) duplexes (*n* = 9) as described in section 2 in the main text (and Fig. 2 legend in the main text). At 7 days, the females were fixed with AFA (48% ethanol, 25% formalin, 2% acetic acid) and stained with Langeron’s Carmine as previously described (Machado-Silva et al., 1998; Geyer et al., 2011). Worms were cleared with xylene and subsequently mounted with DPX (distyrene, plasticizer, xylene) before laser scanning confocal microscopy (LSCM). Damage to the schistosome surface was subjectively quantified by a numerical scale where: 0 = no damage, 1 = minimal damage, 2-3 = localised to considerable damage, 3-4 = considerable to severe damage. Percentages of worms displaying these subjective damage metrics are represented in the stacked histogram. Mid-body regions of two representative worms/treatment are illustrated. White arrows point to surface damage associated with reductions in SmVAL6 transcript/protein levels (see Fig. 2 in the main text). Scale bar = 10 µm. **References:** Geyer, K.K., Rodriguez Lopez, C.M., Chalmers, I.W., Munshi, S.E., Truscott, M., Heald, J., Wilkinson, M.J., Hoffmann, K.F., 2011. Cytosine methylation regulates oviposition in the pathogenic blood fluke *Schistosoma mansoni*. Nat Commun 2, 424. Machado-Silva, J.R., Pelajo-Machado, M., Lenzi, H.L., Gomes, D.C., 1998. Morphological study of adult male worms of *Schistosoma mansoni* Sambon, 1907 by confocal laser scanning microscopy. Mem Inst Oswaldo Cruz 93 Suppl 1, 303-307.

Supplementary figure S3 Incubation of adult worms in a hypotonic solution leads to tegumental barrier disruption. Seven week old female and male schistosomes were either incubated in isotonic (DMEM) or hypotonic (ultrapure water) solutions for 10 min. Afterwards, the worms were labelled with biotin-TAMRA-dextran/Alexa-fluor 488-conjugated phalloidin, processed, fixed and mounted on microscope slides as previously described (Wendt et al., 2018). Mounted worms were then subjected to laser scanning confocal microscopy (LSCM) as described in section 2 in the main text. (A) Representative view of adult female worms (isotonic, n= 3; hypotonic, *n* = 3) together with a box and whisker chart of all collected data (isotonic sections, *n* = 27; hypotonic sections, *n* = 24). (B) Representative view of adult male worms (isotonic, *n* = 3; hypotonic, *n* = 3) together with a box and whisker chart of all collected data (isotonic sections, *n* = 24; hypotonic sections, *n* = 21). Alexa-fluor 488-conjugated phalloidin, green; biotin-TAMRA-dextran, pink (outside) and yellow (inside). Scale bar = 10 µm. **References:** Wendt, G.R., Collins, J.N., Pei, J., Pearson, M.S., Bennett, H.M., Loukas, A., Berriman, M., Grishin, N.V., Collins, J.J., 3rd, 2018. Flatworm-specific transcriptional regulators promote the specification of tegumental progenitors in *Schistosoma mansoni*. Elife 7.
