# Supplementary material for: Schistosoma mansoni venom allergen-like protein 6 (SmVAL6) maintains tegumental barrier function
Source: Int J Parasitol. 2021 Mar;51(4):251–61. doi: 10.1016/j.ijpara.2020.09.004 (PMC7957364; doi:10.1016/j.ijpara.2020.09.004)
Supplement: Supplementary Table S1 [file mmc1.docx]

**Supplementary Table S1.** Reverse transcription quantitative PCR (qRT-PCR), reverse transcription PCR (RT-PCR) and small interfering RNA (siRNA) oligonucleotide sequences used in this study.

| **Common name** | **SchistoGeneDB^a^ designation** | **Oligonucleotide sequences (5´-3´)** | **Product size (bp)** |
| --- | --- | --- | --- |
| **qRT-PCR primers** | | | |
| SmVAL6 | Smp_124050 | GTGAAGAAGTTTCACAGACATGGTATAGT  CAGCTCGTGTTGTAGATTTCCAAATCA | 116 |
| Sm alpha-tubulin | Smp_090120 | CTTCGAACCAGCAAATCAGA  GACACCAATCCACAAACTGG | 157 |
| **RT-PCR primers** | | | |
| Sm14 | Smp_095360.1 | TCTAGAATGTCTAGTTTCTTGGG  CTCGAGTTAGGATAGTCGTTTA | 414 |
| Sm14 DeltaE3 | Smp_095360.2 | TCTAGAATGTCTAGTTTCTTGGG  CTCGAGTTAGGATAGTCGTTTA | 309 |
| **siRNA duplexes** | | | |
| SmVAL6 | Smp_124050 | AUUGAAGUUCUGGACAU  CUACAUGGAUGUCCAGA | n/a |
| Firefly Luciferase | n/a | CUUACGCUGAGUACUUCGA[dT][dT][dT]  UCGAAGUACUCAGCGUAAG[dT][dT][dT] | n/a |

**^a^**Version 7.0

n/a, not applicable
