# Supplementary material for: Schistosoma mansoni venom allergen-like protein 6 (SmVAL6) maintains tegumental barrier function
Source: Int J Parasitol. 2021 Mar;51(4):251–61. doi: 10.1016/j.ijpara.2020.09.004 (PMC7957364; doi:10.1016/j.ijpara.2020.09.004)
Supplement: Supplementary figure S2 — Schistosoma mansoni venom allergen-like protein SmVAL6 reductions impair tegumental barrier function in adult schistosomes. Seven week old female schistosomes were electroporated with either short interfering (si)Smval6 (n = 16) or siLuciferase (siLuc) duplexes (n = 9) as described in section 2 in the main text (and Fig. 2 legend in the main text). At 7 days, the females were fixed with AFA (48% ethanol, 25% formalin, 2% acetic acid) and stained with Langeron’s Carmine as previously described (Machado-Silva et al., 1998; Geyer et al., 2011). Worms were cleared with xylene and subsequently mounted with DPX (distyrene, plasticizer, xylene) before laser scanning confocal microscopy (LSCM). Damage to the schistosome surface was subjectively quantified by a numerical scale where: 0 = no damage, 1 = minimal damage, 2-3 = localised to considerable damage, 3-4 = considerable to severe damage. Percentages of worms displaying these subjective damage metrics are represented in the stacked histogram. Mid-body regions of two representative worms/treatment are illustrated. White arrows point to surface damage associated with reductions in SmVAL6 transcript/protein levels (see Fig. 2 in the main text). Scale bar = 10 µm. References: Geyer, K.K., Rodriguez Lopez, C.M., Chalmers, I.W., Munshi, S.E., Truscott, M., Heald, J., Wilkinson, M.J., Hoffmann, K.F., 2011. Cytosine methylation regulates oviposition in the pathogenic blood fluke Schistosoma mansoni. Nat Commun 2, 424. Machado-Silva, J.R., Pelajo-Machado, M., Lenzi, H.L., Gomes, D.C., 1998. Morphological study of adult male worms of Schistosoma mansoni Sambon, 1907 by confocal laser scanning microscopy. Mem Inst Oswaldo Cruz 93 Suppl 1, 303-307. [file mmc2.pdf]

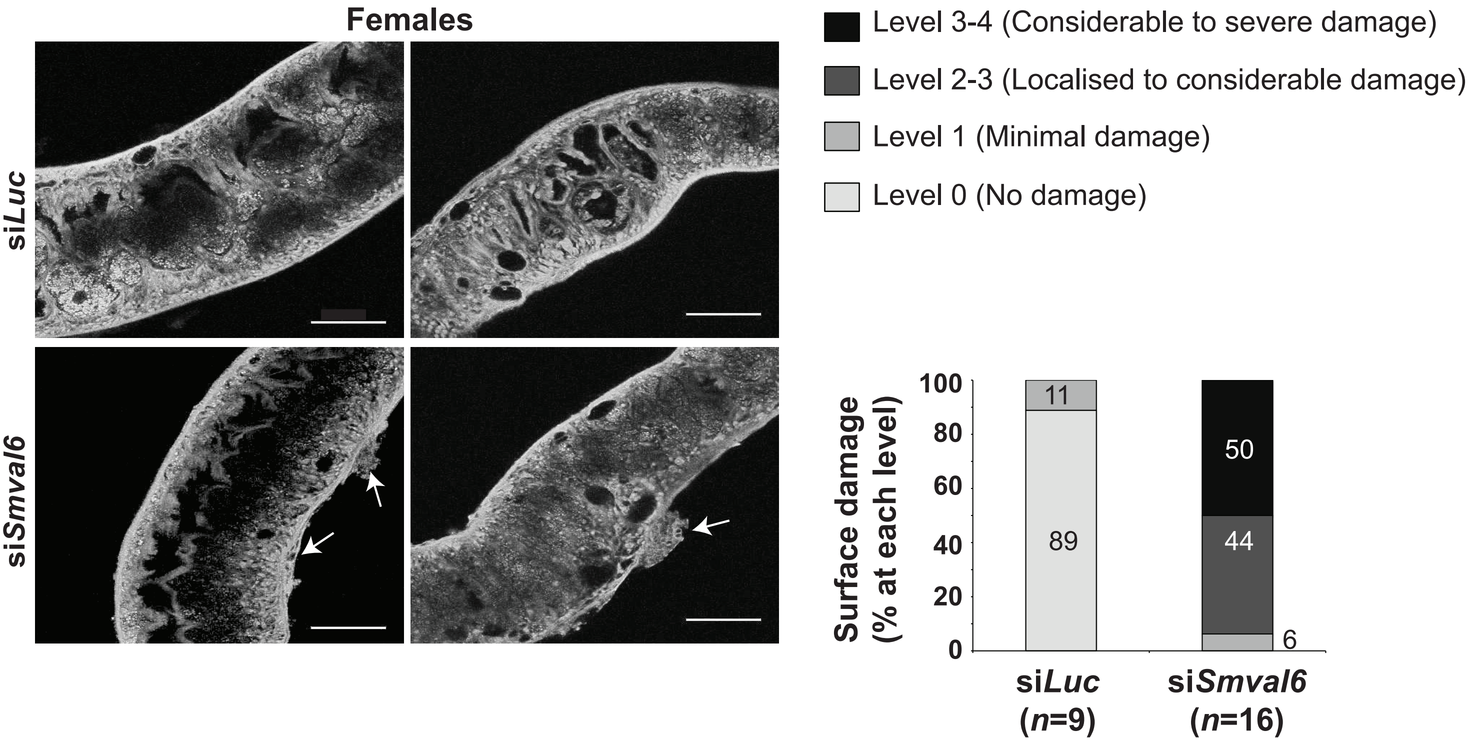
Females
siLuc
siSmval6
Level 3-4 (Considerable to severe damage)
Level 2-3 (Localised to considerable damage)
Level 1 (Minimal damage)
Level 0 (No damage)
Surface damage (% at each level)
0
20
40
60
80
100
11
89
50
44
6
siLuc (n=9)
siSmval6 (n=16)
