# Supplementary figures and images for: Schistosoma mansoni venom allergen-like protein 6 (SmVAL6) maintains tegumental barrier function
Source: Int J Parasitol. 2021 Mar;51(4):251–61. doi: 10.1016/j.ijpara.2020.09.004 (PMC7957364; doi:10.1016/j.ijpara.2020.09.004)

A

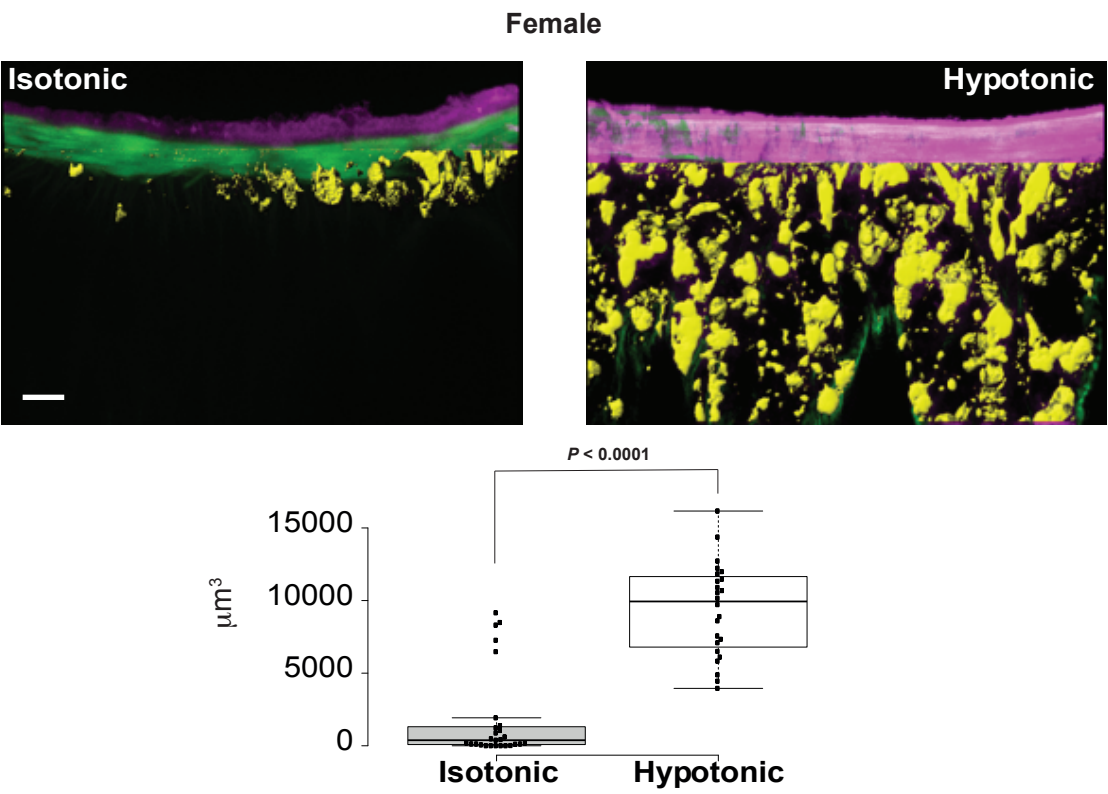

B

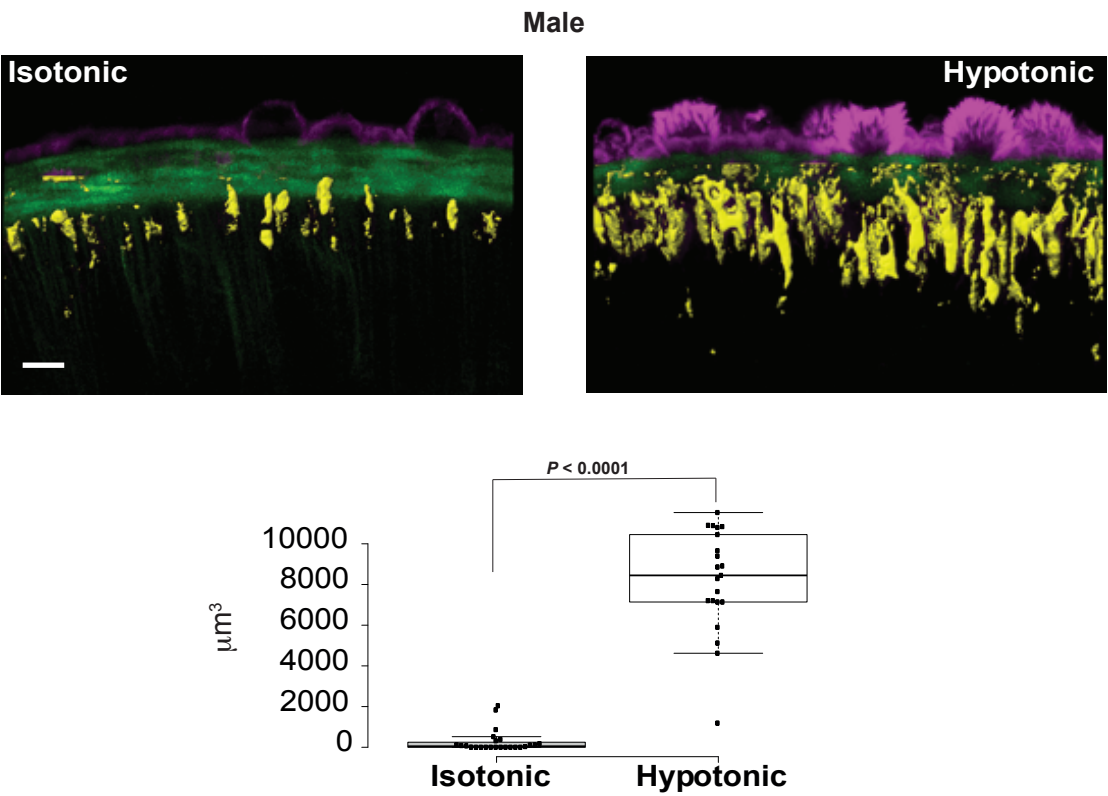

Supplement: Supplementary figure S3 — Incubation of adult worms in a hypotonic solution leads to tegumental barrier disruption. Seven week old female and male schistosomes were either incubated in isotonic (DMEM) or hypotonic (ultrapure water) solutions for 10 min. Afterwards, the worms were labelled with biotin-TAMRA-dextran/Alexa-fluor 488-conjugated phalloidin, processed, fixed and mounted on microscope slides as previously described (Wendt et al., 2018). Mounted worms were then subjected to laser scanning confocal microscopy (LSCM) as described in section 2 in the main text. (A) Representative view of adult female worms (isotonic, n= 3; hypotonic, n = 3) together with a box and whisker chart of all collected data (isotonic sections, n = 27; hypotonic sections, n = 24). (B) Representative view of adult male worms (isotonic, n = 3; hypotonic, n = 3) together with a box and whisker chart of all collected data (isotonic sections, n = 24; hypotonic sections, n = 21). Alexa-fluor 488-conjugated phalloidin, green; biotin-TAMRA-dextran, pink (outside) and yellow (inside). Scale bar = 10 µm. References: Wendt, G.R., Collins, J.N., Pei, J., Pearson, M.S., Bennett, H.M., Loukas, A., Berriman, M., Grishin, N.V., Collins, J.J., 3rd, 2018. Flatworm-specific transcriptional regulators promote the specification of tegumental progenitors in Schistosoma mansoni. Elife 7. [file mmc3.pdf]
